# Identification and mitigation of unexplained dose attenuation caused by sub‐table water accumulation in an MR‐Linac (Elekta Unity)

**DOI:** 10.1002/acm2.70441

**Published:** 2025-12-29

**Authors:** Jinhu Chen, YuKun Li, Xingwei An, Zhenjiang Li

**Affiliations:** ^1^ Academy of Medical Engineering and Translational Medicine Tianjin University Tianjin China; ^2^ Department of Radiation Oncology Physics and Technology Shandong Cancer Hospital and Institute Shandong First Medical University and Shandong Academy of Medical Sciences Jinan China

**Keywords:** dose attenuation, elekta unity, environmental control, MR‐Linac, quality assurance, root cause analysis

## Abstract

**Purpose:**

To report and investigate a case of significant dose attenuation observed during quality assurance (QA) of an Elekta Unity MR‐Linac system, and to elucidate its root cause and solution.

**Methods:**

During a period of abnormal environmental conditions (elevated temperature and humidity), a >20% dose output reduction was detected at a gantry angle of 180°. A comprehensive investigation was undertaken, including dose output measurements at multiple gantry angles, beam quality assessment, mechanical accuracy checks, EPID image analysis, and gamma pass rate analysis using ArcCheck‐MR. The source of the attenuation was localized through a process of elimination.

**Results:**

The investigation revealed severe dose attenuation specifically at angles traversing beneath the treatment couch, with a gamma pass rate (3%/3 mm) dropping to 39.1% at 180°. All other system parameters, including MRI quality and mechanical alignments, were within tolerance. Visual inspection after disassembly confirmed the presence of a substantial pool of water beneath the table, estimated to be at least 3.5 cm deep, which acted as an unintended attenuator. The water was attributed to condensation and drainage issues exacerbated by the failed air‐conditioning system. Mitigation strategies, including hardware sealing, enhanced drainage, revised maintenance protocols, and the addition of a 180° dose check to daily QA, successfully resolved the issue.

**Conclusion:**

This case highlights a previously unreported and critical failure mode for MR‐Linac systems. It underscores the profound impact of environmental control on machine performance and patient safety. Robust HVAC maintenance, proactive monitoring of sub‐table areas, and the inclusion of oblique‐angle dosimetry in QA routines are essential for all Elekta Unity and similar high‐precision radiotherapy systems.

## 1 INTRODUCTION

The integration of magnetic resonance imaging with a linear accelerator (MR‐Linac), as exemplified by the Elekta Unity system, represents a significant advancement in precision radiotherapy. This technology enables real‐time soft‐tissue visualization and on‐table adaptive therapy, facilitating highly conformal dose delivery to targets while sparing surrounding healthy organs.^1^, ^2^ However, the convergence of an MRI scanner and a linear accelerator within a single system introduces unprecedented complexity, necessitating a more sophisticated and rigorous quality assurance (QA) framework than conventional Linacs.^3^, ^4^

The QA for the Elekta Unity must address unique challenges stemming from its hybrid design. The presence of a 1.5 T magnetic field influences the trajectory of secondary electrons, altering dose deposition characteristics and requiring specialized beam modeling and commissioning.^5^, ^6^ Furthermore, the mechanical cohabitation of the two systems demands exquisite accuracy in the coincidence of the radiation and imaging isocenters.^7^ Consequently, comprehensive QA protocols encompassing dosimetry, mechanical integrity, and image quality have been established by the community and consortium guidelines to ensure ongoing system performance and patient safety.^8^, ^9^ Although these protocols thoroughly address the system's internal interactions, reports on the impact of external environmental factors on the physical hardware and subsequent dosimetric output remain scarce. Most guidelines monitor ambient temperature and humidity for system stability but seldom document instances where environmental failures directly lead to critical, hidden hardware issues causing major dosimetric errors.

This study reports a rare but critical safety incident of significant dose attenuation caused by an unexpected root cause: water accumulation beneath the treatment table of an Elekta Unity system, subsequent to a failure of the room's air conditioning unit. We detail the systematic investigation that localized the fault, the root cause analysis, and the effective mitigation strategies implemented. The purpose of this report is to alert the community to this potential failure mode and to underscore the importance of robust environmental control and proactive maintenance in safeguarding the operational integrity of high‐precision radiotherapy equipment.

## 2 MATERIALS AND METHODS

### 2.1 Initial problem identification and environmental context

The incident occurred against a backdrop of a critical failure in the treatment room's air conditioning system. It is critical to note that this HVAC system is an independent infrastructure component, separate from the Elekta Unity machine itself, responsible for maintaining the entire vault's ambient conditions. Over a period of several days (July 14–19, 2022), the room temperature progressively increased from 24°C to a peak of 31°C, while the relative humidity rose from 70% to 95%. We hypothesize that this extreme environment led to massive condensation forming on the cooler internal mechanical components of the MR‐Linac system. During the initial phase of this environmental disturbance, patient treatments continued, and patient‐specific quality assurance (QA) for adapted plans showed largely acceptable results (gamma pass rate ≥93.3%, 3%/3 mm). However, on July 23rd, after the air conditioning had been restored, a routine machine QA check revealed a severe dose output attenuation of more than 20% specifically at a gantry angle of 180°. This finding triggered a comprehensive investigation. A chronological timeline of the key events, from the initial HVAC failure to the final resolution, is summarized in Table 1 to provide a clear overview of the incident sequence.

**TABLE 1:** Timeline of key events.

Date	Event / Phase	Description	Figure
14 July 2022 to 19 July 2022	HVAC failure and environmental deterioration	A critical failure of one compressor in the room's air‐conditioning system led to a progressive deterioration of the ambient environment: temperature rose from 24°C to 31°C, and relative humidity increased from 70% to 95%. The engineering team was notified for repair.	
14 July 2022 to 22 July 2022	Continued clinical operations	Patient treatments continued during this period. Patient‐specific QA for adapted plans showed acceptable results (gamma pass rate ≥ 93.3%, 3%/3 mm), failing to detect the latent hardware issue.	
23 July 2022	Problem identification and treatment halt	After the HVAC system was restored, a routine machine QA check revealed a severe dose output attenuation (>20%) specifically at a gantry angle of 180°. This finding triggered an immediate halt to all patient treatments and initiated a formal investigation.	
24 July 2022	Initial investigation (Linac Side)	The investigation began with the disassembly of the linac components (gun side). No obstruction or abnormality was found, ruling out issues from the radiation source forward.	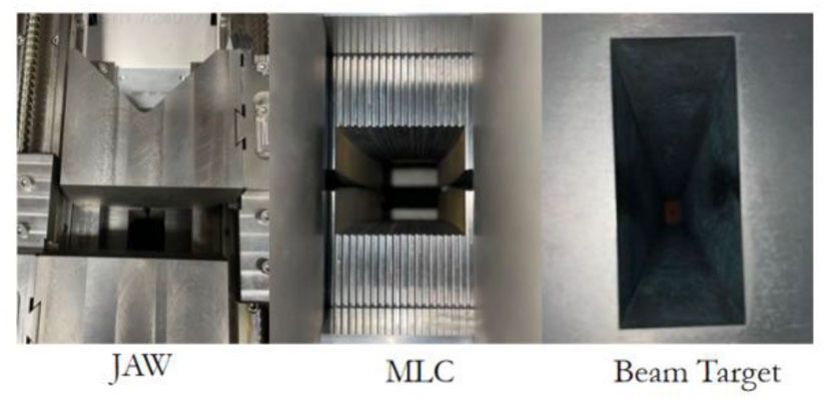
25 July 2022	Investigation (Couch Assembly)	The investigation moved to the treatment couch assembly. Again, no anomalies were identified, narrowing down the potential source of attenuation.	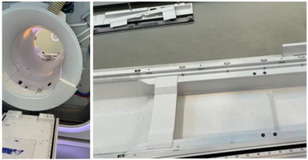
26 July 2022	Root cause identified	The investigation focused on the subcouch area. Upon removal of the body coil shell, a substantial accumulation of clear water was visually identified and confirmed as the unintended physical attenuator.	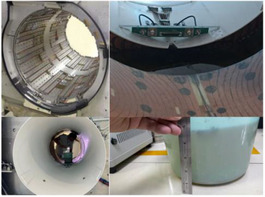
27 July 2022	Mitigation and system remediation	The accumulated water was removed. The area was thoroughly dried. To ensure immediate drainage and monitor for any recurrence, a temporary syringe‐based drainage port was installed as an interim measure.	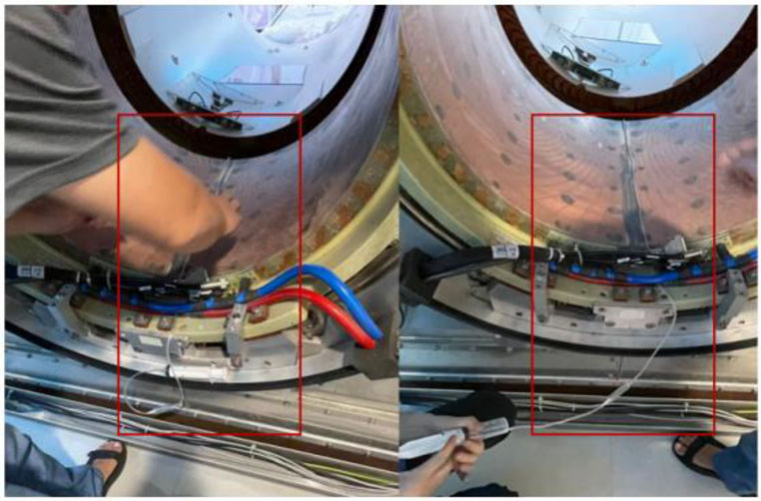
28 July 2022 to 29 July 2022	System verification and quality assurance	A comprehensive series of QA tests, including dosimetric output, beam profile, and gamma analysis, were performed. All results confirmed that the system performance had returned to within established tolerances.	
30 July 2022 and beyond	Resumption of clinical operations and long‐term measures	Clinical treatments resumed. Long‐term mitigation strategies, including enhanced sealing, improved drainage design, revised preventive maintenance protocols, and the addition of a 180° dose check to the daily QA, were implemented.	

### 2.2 Comprehensive Linac QA tests

To rule out common causes of dosimetric inaccuracy, a series of standard QA tests were performed on the Elekta Unity system:

1. Absolute dosimetry: Dose output was measured at four cardinal gantry angles (0°, 90°, 180°, and 270°) using an ionization chamber.
2. Beam quality: The beam quality index (TPR20/10) was evaluated to check for any beam energy shifts.
3. Mechanical accuracy: The accuracy of the couch travel position, jaw position, and gantry rotation were assessed against baseline tolerances.
4. EPID image quality: The performance of the Electronic Portal Imaging Device (EPID) was analyzed using standard tests.^10^
5. MRI quality: Periodic image quality testing (PIQT) of the magnetic resonance imaging (MRI) system, including MR scaling and MR‐to‐MV isocentric coincidence, was conducted.

### 2.3 Localization of the attenuation source

To precisely characterize the nature and extent of the dose attenuation, targeted investigations were conducted:

1. Point dose measurements: We have specified that measurements were performed for a 10 cm × 10 cm field, at a depth of 10 cm in water, with an SSD of 133.5 cm, using a PTW 30013 ionization chamber. The results are now presented as mean ± standard deviation, demonstrating high reproducibility (standard deviation <0.5%) at nonattenuated angles.
2. Gamma analysis: We have explicitly defined the analysis criteria. Although the original text mentioned the “3%/3 mm criterion and a 10% global threshold,” we have reiterated this for clarity and specified that the analysis was performed using the SNC Patient software.
3. Dose output profiling: Dose output was measured in detail across 17 gantry angles (from 100° to 260°) to map the angular dependence of the attenuation.
4. 3D dosimetry verification: A volumetric gamma analysis was performed using the ArcCheck‐MR diode array (Sun Nuclear Corporation). A 10 cm × 10 cm standard field was delivered at 35 gantry angles (spaced ∼10° apart), and the resulting dose distribution was analyzed using SNC Patient software with a 3%/3 mm criterion and a 10% global threshold^11^
5. Couch position dependency: Key measurements (e.g., dose output) were repeated with the phantom positioned at three different locations along the couch travel: isocenter, head end, and foot end, to determine if the effect was localized.
6. EPID imaging for obstruction: EPID images of a large field (22 cm × 22 cm) were acquired at gantry angles of 0°, 150°, 180°, and 210° to visually inspect for any physical obstructions in the beam's path.

### 2.4 Root cause investigation

When the above tests localized the problem to the region beneath the treatment couch, a physical inspection was initiated. The machine was partially disassembled with the assistance of field service engineers from Elekta and Philips. Initial inspection of the area forward of the tungsten target revealed no obstructions. The investigation then focused on the sub‐table area. The backward coil was removed and ruled out as the cause. Finally, upon removal of the body coil shell, a substantial accumulation of clear water was visually identified and confirmed to be the attenuating material (see Figure 4a/5). This finding conclusively identified the root cause.

## 3 RESULTS

### 3.1 Clinical impact overview

During the period of undetected machine abnormality, a total of 23 patients underwent 74 treatment fractions. Subsequent analysis identified that 81 treatment fields were delivered with the potential dose attenuation, corresponding to a cumulative monitor unit count of 27 388.85 MU. The distribution of affected fractions per patient is summarized in Table 2.

**TABLE 2:** The number of fractions and fields of the affected patients.

Patient	Fraction	Dose(Gy)	Number of affected fields(n)	Total fields	Affected MU	Total MU
Patient 1	2	3	3	6	246.59	489.86
				6	258.96	477.49
Patient 2	2	2.5	5	8	270.49	431.87
				8	275.54	440.9
Patient 3	4	2	2	8	60.41	465.16
				8	60.32	457.73
				8	64.3	467.54
				8	59.21	459.57
Patient 4	3	3	3	7	367.68	954.64
				7	357.39	964.8
				7	403.67	976.38
Patient 5	4	3	5	8	573.09	889.71
				8	616.15	919.73
				8	580.67	897.38
				8	585.3	883.04
Patient 6	4	5	3	8	331.19	1033.07
				8	318.6	986.74
				8	290.74	1035.12
				8	291.87	967.43
Patient 7	4	5	0	7	0	983.22
Patient 8	3	9	5	11	1009.76	1919.26
				11	1334.42	2138.37
				11	1096.41	1897.89
Patient 9	4	5	7	7	1163.38	1163.38
				7	1114.71	1114.71
				7	1145.05	1145.05
				7	1145.9	1145.9
Patient 10	4	1.8	1	7	54.28	387.93
				7	43.95	380.88
				7	44.7	385.59
				7	49.68	379.63
Patient 11	2	2	2	7	267.76	678.66
				7	268.68	673.45
Patient 12	4	2.4	0	7	0	493.71
Patient 13	3	3	1	6	188.77	739.52
				6	201.13	731.56
				6	190.08	765.65
Patient 14	4	2	4	8	211.82	408.24
				8	209.85	409.81
				8	213.03	418.31
				8	202.77	403.63
Patient 15	4	2.2	3	8	269.12	882.79
				8	280.35	879.8
				8	273.03	866.44
				8	274.37	879.61
Patient 16	4	3	3	9	563.41	1307.31
				9	548.28	1293.6
				9	485.45	1207.73
				9	540.37	1278.7
Patient 17	3	8	5	10	1024.65	1800.59
				10	1023.78	1807.43
				10	997.57	1786.48
Patient 18	4	2.4	3	9	382.21	1192.16
				9	373.36	1186.71
				9	380.42	1209.95
				9	381.03	1214.17
Patient 19	4	2.2	3	9	250.8	796.54
				9	243.7	791.35
				9	249.28	789.5
				9	237.18	793.66
Patient 20	2	8	1	10	53.29	1370.19
			1	10	27.6	1360.91
Patient 21	2	8	5	10	537.37	1444.56
			5	10	525.99	1433.44
Patient 22	2	1.8	4	8	305.61	563.37
			4	8	307.93	556.66
Patient 23	2	2	3	9	359.18	1009.04
			3	9	325.22	961.61

We specify that the reported 27 388.85 MU refers to the Monitor Units delivered specifically at the affected gantry angles (primarily around 180°, about 160°–200°).

### 3.2 Dosimetric impact on a representative patient

**FIGURE 1:**
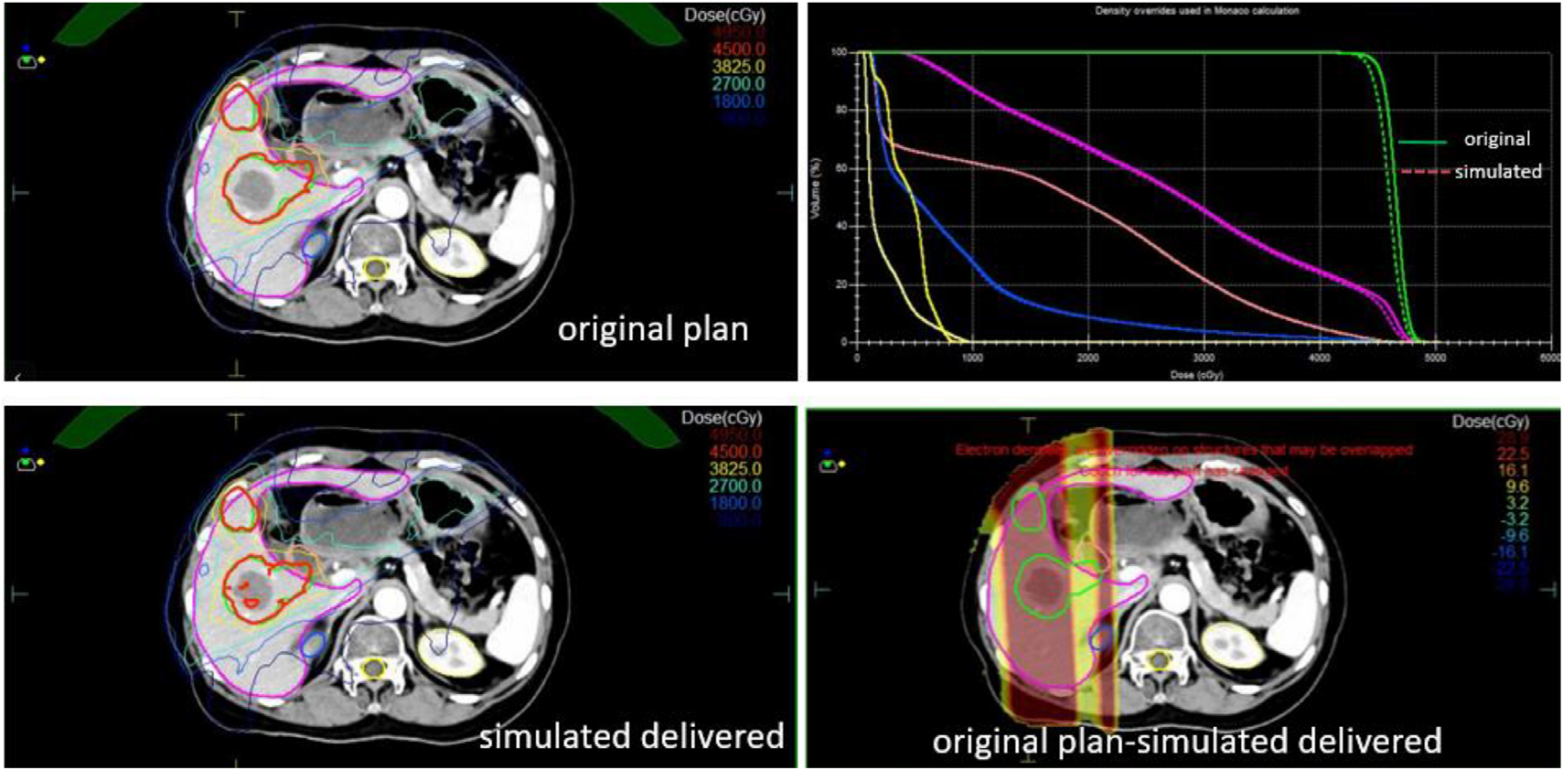
Comparison of planned and delivered (simulated) dose distributions and DVHs for a representative pelvic patient.

The comparison between the original and the “simulated delivered” dose distributions revealed that the planning target volume (PTV) D95 (dose to 95% of the volume) was reduced by approximately 1.9%. Furthermore, there was a non‐negligible increase in dose to some organs at risk (e.g., liver and kidney). The detailed results were shown in Figure 1.

### 3.3 Comprehensive QA findings: A singular anomaly

The systematic QA investigation yielded a critical finding: all standard system parameters were within established clinical tolerances, except for the severe dose attenuation. Specifically:

1. Beam quality (TPR20/10) remained stable.
2. Mechanical accuracies for the couch, jaws, and gantry were within 0.5 mm.
3. MRI system performance, including image quality and MR‐to‐MV isocentric coincidence, passed all periodic tests.
4. EPID image uniformity showed no evidence of obstruction in the beam's path from the gun side.

### 3.4 Characterization of the dose attenuation

The dosimetric measurements unequivocally localized the problem to the region beneath the treatment couch.

**FIGURE 2:**
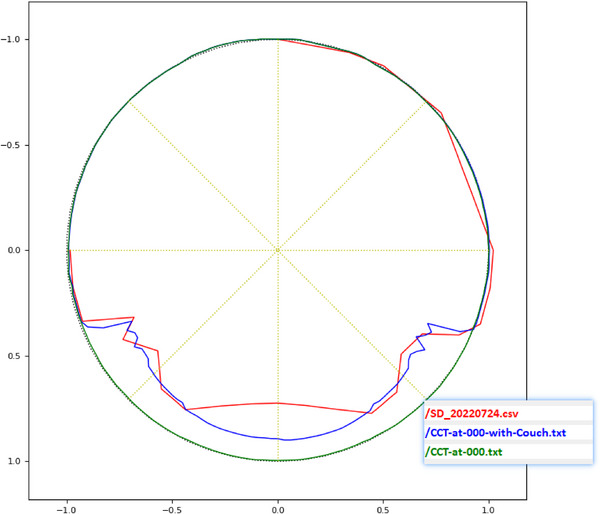
Figure illustrates the measurement results across the 17 gantry angles, with the measurements depicted by the red line. The blue line is the result of the commission measurement.

**FIGURE 3:**
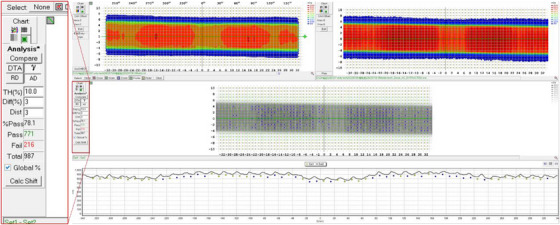
The results of gamma pass rate analysis for 35 fields.

1. Point dose measurement: A severe dose output attenuation of approximately 30% was confirmed at a gantry angle of 180° (Table 3).
2. Angular dependence profile: Detailed profiling across 17 gantry angles revealed that the attenuation was exclusively pronounced when the beam was directed through the subcouch area, with output returning to normal outside this arc (Figure 2).
3. Volumetric verification: This finding was corroborated by the ArcCheck‐MR gamma analysis. Although the pass rate (3%/3 mm) was 100% at 0°, it plummeted to 39.1% at 180°. The pass rate for a full arc of 35 fields was significantly reduced to 78.1% (Figure 3, Table 4).

**TABLE 3:** Two dose output measurements before and after the accident.

2022.7.23	Depth 10 cm 100MU field size 10 cm×10 cm				Depth 20 cm 100MU field size 10 cm×10 cm	TPR20/10
	G0(mGy)	G90(mGy)	G180(mGy)	G270(mGy)	G0(mGy)	
Head of table	856.6	848.8	587.6	850.1	605	0.706
Middle of table			587.5			
Bottom of table			587.8			
2022.7.08	Depth 10 cm 100MU field size 10 cm×10 cm				Depth 20 cm 100MU field size 10 cm×10 cm	TPR20/10
	G0(mGy)	G90(mGy)	G180(mGy)	G270(mGy)	G0(mGy)	
Head of table	854.6	850.1	753.5	851.3	605.6	0.709

**TABLE 4:** Gamma pass rate analysis results.

	Unity			
	Local		Global	
PlanID	DTA(%)	Gamma(%)	DTA(%)	Gamma(%)
G180	31.2	32.4	38.4	39.1
G150	90.6	90.5	99.3	99.2
G0	93.3	93.4	100.0	100.0
F_35filed	65.2	65.2	78.1	78.1

### 3.5 Identification of the root cause

**FIGURE 4:**
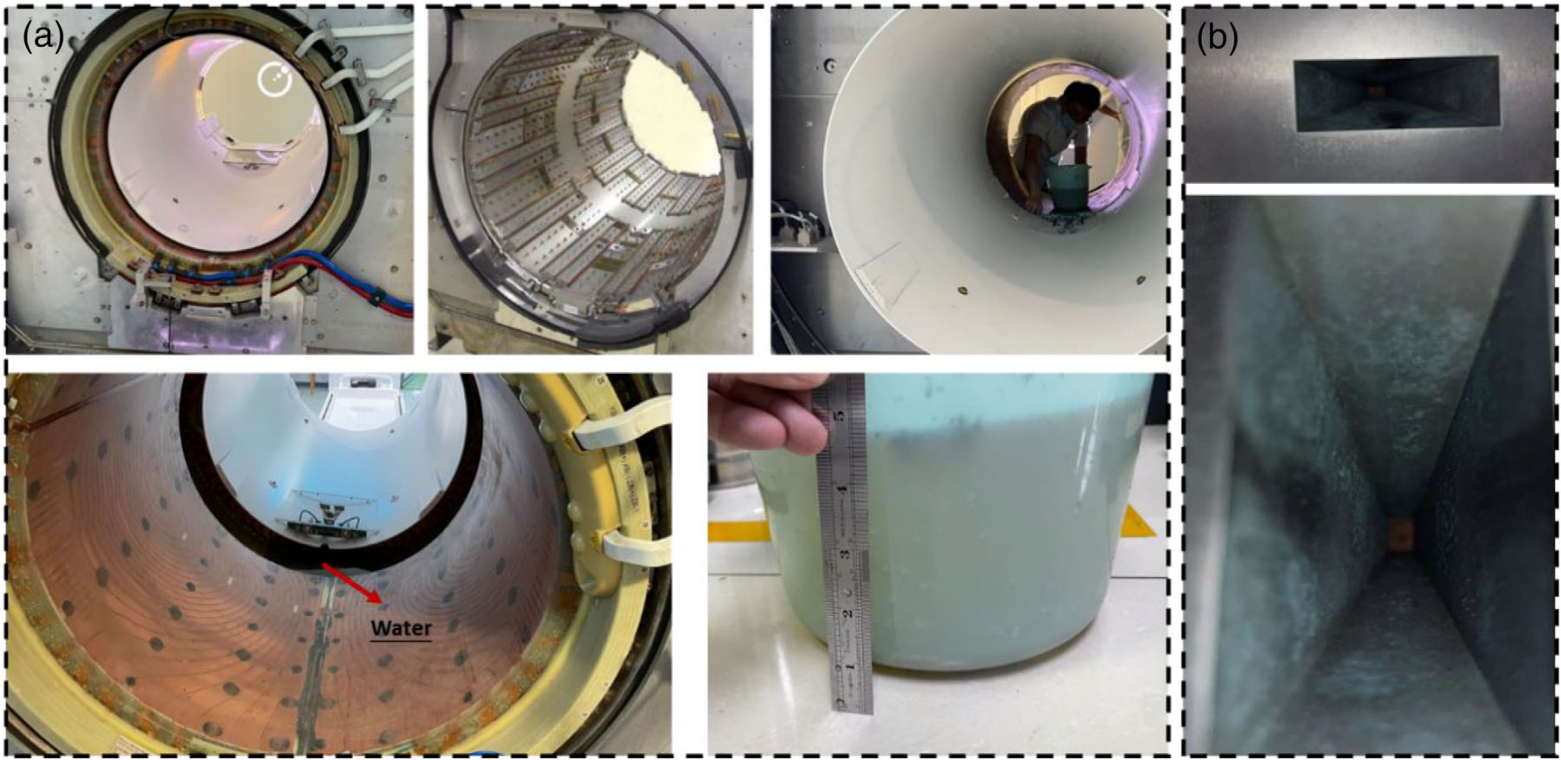
Figure A depicts the detailed disassembly process, allowing for a clear view of the water's location. Figure B demonstrates that no obstruction is present in front of the tungsten target.

The physical inspection, guided by the dosimetric evidence, conclusively identified the root cause. Upon disassembly of the subcouch components, a substantial accumulation of water was visually identified and confirmed (Figure 4a/5). No other obstructions were found in the beam path (Figure 4b).

**FIGURE 5:**
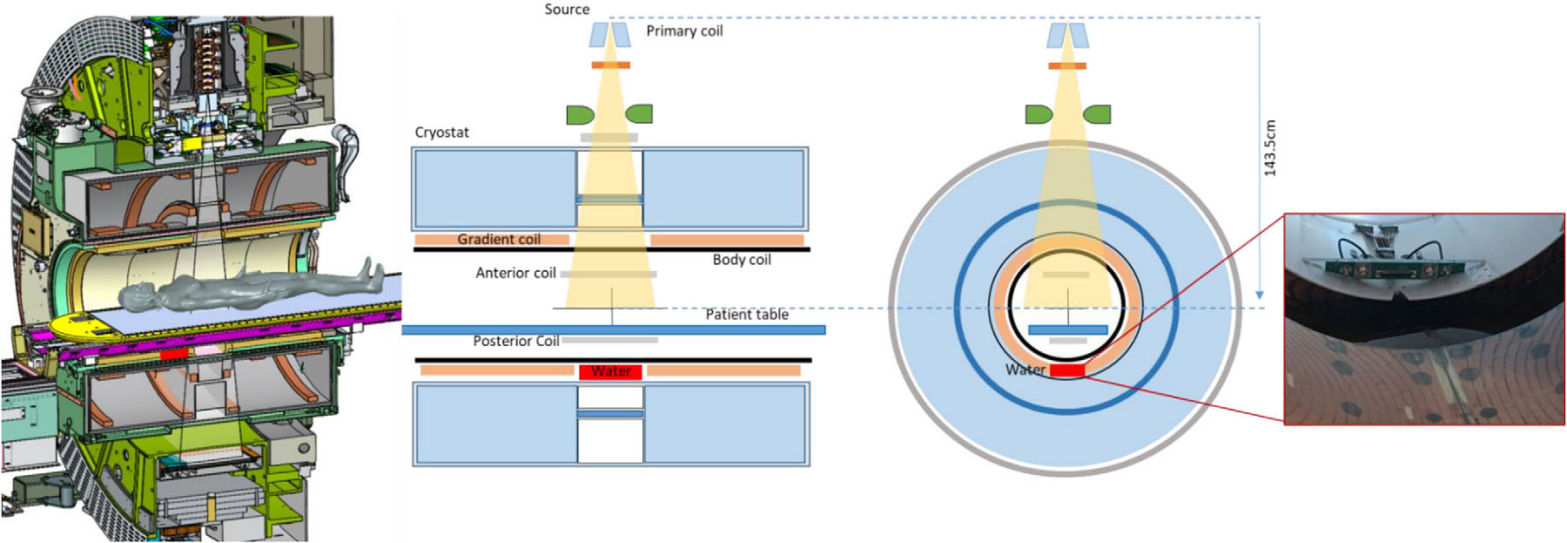
Figure shows the location of the water in detail.

## 4 DISCUSSION

This incident elucidates a critical and previously unreported failure mode for the Elekta Unity MR‐Linac, where water accumulation beneath the treatment couch was identified as the root cause of significant and localized dose attenuation. Our systematic investigation confirmed that a pool of water, estimated to be at least 3.5 cm deep, acted as an unintended physical attenuator. The observed ∼30% dose reduction at 180° is consistent with the known attenuation properties of water for megavoltage x‐rays, providing a solid physical basis for our findings.

The origin of this water is strongly correlated with the preceding failure of the treatment room's air conditioning system. The prolonged period of elevated temperature and extreme humidity (up to 31°C and 95% RH) created conditions ripe for substantial condensation within the machine's enclosed structure. This chain of events underscores a critical vulnerability: a failure in the room's independent environmental infrastructure can precipitate a hidden hardware fault (water accumulation) within the radiotherapy device, ultimately compromising its dosimetric accuracy. Although other sources were considered, the temporal link to the environmental breakdown makes condensation the most plausible primary source. This chain of events underscores a direct and alarming pathway through which a failure in room infrastructure can precipitate a critical hardware fault, ultimately compromising dosimetric accuracy.

A particularly disconcerting aspect of this event was its evasion of detection by patient‐specific QA, which proceeded with acceptable gamma pass rates during the initial period of machine abnormality. This highlights a fundamental limitation: patient‐specific plan verification, often performed at a single reference gantry angle, is designed to validate the treatment planning process and not to comprehensively audit the machine's output across all possible clinical angles. Consequently, 23 patients underwent 74 treatment fractions under a systematic error, representing a serious patient safety incident with potential for tumor under‐dosage and organ‐at‐risk over‐exposure. This starkly reinforces the indispensable role of robust, independent machine QA as the primary defense against such device‐specific failures.

Following the discovery of this incident, an immediate clinical risk assessment and root cause analysis were initiated. All affected patients underwent detailed dose reconstruction and clinical evaluation. In accordance with our institution's quality management system and national regulatory requirements, this was classified as a reportable medical event. In collaboration with the responsible oncologists, all affected patients were individually informed, and the situation was transparently explained, including its potential impact on their treatment. For a subset of patients where the dose deviation was deemed to have potential clinical significance, compensatory re‐planning and additional treatment fractions were offered, and they were enrolled into a dedicated follow‐up pathway.

This case serves as a crucial alert for all centers operating similar high‐precision radiotherapy equipment. The reliability of environmental control systems must be treated as a cornerstone of patient safety, especially in climates prone to high humidity. To mitigate this specific risk, we advocate for the integration of a simple point dose measurement at a gantry angle of 180° into the daily QA routine—a highly efficient test capable of intercepting this failure mode. Furthermore, we recommend instituting periodic visual inspections of the subcouch area as a mandatory component of preventive maintenance schedules. We acknowledge the limitations of this study as a single‐institution case report, and the precise quantification of the water accumulation rate remains uncertain. Furthermore, upon sharing this incident with the manufacturer (Elekta), we understand that they have subsequently issued technical guidance to their global user base regarding this issue and have considered relevant design improvements in subsequent equipment productions.

In conclusion, this experience delivers a powerful lesson: environmental control is not merely a matter of comfort but an integral component of the dosimetric chain for advanced radiotherapy systems. It compellingly argues for the establishment of a multilayered, defense‐in‐depth quality assurance strategy that rigorously integrates environmental monitoring, comprehensive machine QA, and proactive physical inspections. For technologies like the MR‐Linac to reliably deliver their promised precision, the foundational systems ensuring their basic operational integrity must be upheld with the utmost vigilance.

## AUTHOR CONTRIBUTIONS

**Zhenjiang Li**: Conceptualization; methodology; writing‐reviewing and editing; validation. **Jinhu Chen**: Original draft preparation. **Yukun Li**: Investigation; data curation.

## CONFLICT OF INTEREST STATEMENT

The authors declare no conflicts of interest.

## ETHICAL APPROVAL AND CONSENT TO PARTICIPATE

This study was reviewed and approved by the Institutional Review Board of Shandong Cancer Hospital. All methods of this study were carried out in accordance with the relevant guidelines and regulations.

## Data Availability

Data is provided within the manuscript.
